# Spontaneous Bacterial Peritonitis among Chronic Liver Disease Patients with Ascites Admitted to the Department of Medicine of a Tertiary Care Centre: A Descriptive Cross-sectional Study

**DOI:** 10.31729/jnma.8124

**Published:** 2023-04-30

**Authors:** Tarkeshwor Mahato, Yubaraj Sharma, Suman Thapa

**Affiliations:** 1 Department of Internal Medicine, Madhesh Institute of Health Science, Manimandap, Janakpurdham, Nepal; 2 Department of Internal Medicine, Patan Academy of Health Sciences, Lagankhel, Lalitpur, Nepal

**Keywords:** *ascites*, *liver diseases*, *peritonitis*, *prevalence*

## Abstract

**Introduction:**

Chronic liver disease is a common problem worldwide. Spontaneous bacterial peritonitis is a dreaded complication and has high in-hospital mortality. Few studies have been done about the prevalence of spontaneous bacterial peritonitis and associated clinical and biochemical features in a hospital-based population. The aim of this study was to find out the prevalence of spontaneous bacterial peritonitis in chronic liver disease patients with ascites admitted to Department of Medicine in a tertiary care centre.

**Methods:**

A descriptive cross-sectional study was done among patients with chronic liver disease with ascites admitted to the Department of Medicine of a tertiary care centre between 18 March 2021 to 28 February 2022 after receiving ethical approval from the Institutional Review Committee (Reference number: PMM2103161493). Convenience sampling method was used. Diagnostic paracentesis was done in every such patient. Point estimate and 95% Confidence Interval were calculated.

**Results:**

Among 157 patients, the prevalence of spontaneous bacterial peritonitis was 46 (29.29%) (22.17-36.41, 95% Confidence Interval). The most common presenting symptom was pain abdomen seen in 29 (63.04%).

**Conclusions:**

The prevalence of spontaneous bacterial peritonitis in chronic liver disease patients with ascites was similar to studies done in similar settings. Clinicians should be aware that it can present with or without abdominal pain.

## INTRODUCTION

Cirrhosis and chronic liver disease (CLD) are the leading cause of mortality and morbidity.^1^ Spontaneous Bacterial Peritonitis (SBP) is a bacterial infection of ascitic fluid, without a specific identifiable intra-abdominal treatable source of infection.^2^ In hospitalized cirrhotic patients worldwide, SBP can occur in up to 30%.^3^ In studies done in Punjab, India and Dharan, Nepal, the prevalence was 20.4% and 15.2% respectively.^4,5^

Patients can be asymptomatic or have local symptoms, such as abdominal pain. Therefore, diagnostic paracentesis should be performed in all patients with cirrhosis and ascites on admission.^6^ Risk factors are low ascitic fluid protein, impaired renal function, liver failure, upper gastrointestinal bleeding and history of SBP.^7,8^ Early diagnosis increases survival and prevents complications.

The aim of this study was to find out the prevalence of spontaneous bacterial peritonitis in chronic liver disease patients with ascites admitted to Department of Medicine in a tertiary care centre.

## METHODS

A descriptive cross-sectional study was done among patients of CLD with ascites admitted at the Department of Medicine in Patan Hospital. Ethical approval was obtained from the Institutional Review Committee of Patan Academy of Health Sciences (Reference number: PMM2103161493). Data were collected between 18 March 2021 to 28 February 2022. All CLD patients with ascites who were admitted to the Department of Medicine for any indication with an age >18 years, giving consent were included in the study. Patients with secondary peritonitis, who refused consent and with hepatic encephalopathy whose family members did not give consent were excluded from the study. Chronic liver disease was defined by the patient with clinical features suggestive of decompensated liver disease such as ascites, jaundice and/or hepatic encephalopathy; and ultrasound findings of coarse echotexture and/or nodular surface of the liver and a history of duration of illness of more than 6 months. Informed consent was signed and confidentiality of the information was ensured. A convenience sampling method was used. The sample size was calculated by using the following formula:


$$\begin{array}{ll} \text{n} & ={\text{Z}}^{2}\times \frac{\text{p}\times \text{q}}{{\text{e}}^{\text{2}}}\\ & =1.96^{2}\times \frac{0.50\times 0.50}{0.08^{2}}\\ & =151 \end{array}$$

Where,

n = minimum required sample size
Z = 1.96 at 95 % Confidence Interval (CI)
p = prevalence taken as 50% for maximum sample size calculation
q = 1-p
e = margin of error, 8%

The calculated minimum required sample size was 151. However, 157 patients were included in the study.

Data was collected on a predetermined proforma covering the relevant subjects of the study. Each patient was subjected to detailed history taking, physical examination and investigations. Abdominal paracentesis was done with aseptic precautions, bedside ultrasound was used when required; in each and every patient at the time of admission for the first time either in the emergency room, outpatient department or ward by the treating physician. Ascitic fluid was sent for culture and sensitivity and for testing total leukocyte count, differential leukocyte count, total protein, albumin, sugar and fluid Gram staining. Complete blood count, liver function test including total protein, serum albumin and prothrombin time/ international normalised ratio (PT/INR), renal function test, ultrasonography of abdomen and pelvis and additionally in all newly diagnosed chronic liver disease patient and/or those with hematemesis or melena, upper gastrointestinal endoscopy was also done.

SBP was diagnosed based on absolute neutrophil count (ANC) in ascitic fluid ≥250 cells/mm^3^ and/or positive ascitic fluid culture and classified accordingly as classical SBP (CSB), culture-negative neutrocytic ascites (CNNA) and monomicrobial non-neutrocytic bacterascites (MNB). Classical SBP was defined by the presence of ascitic fluid ≥250 cells/mm^3^ and positive ascitic fluid culture. Culture-negative neutrocytic ascites existed when the ascitic fluid culture results were negative, but the PMN count was ≥250 cells/ml. Monomicrobial non-neutrocytic bacterascites existed when a positive culture result coexisted with a neutrophil count <250 cells/ml.^9^

Hepatic encephalopathy was diagnosed by history and physical examination to detect the cognitive dysfunction as confusion, reversed sleep awake cycle and neuromuscular impairments such as flapping tremor that characterize hepatic encephalopathy and exclusion of other causes of mental status changes such as metabolic abnormalities as hyponatremia. Fever was diagnosed by morning 6 a.m. temperature of >37.2° C (>98.9° F) or evening 4-6 p.m. temperature of >37.7° C (>99.9° F).^3^

Abdominal pain was defined as an unpleasant sensation localized to the abdominal region.^3^ According to the International club of ascites, moderate ascites were diagnosed by moderate symmetrical distension of the abdomen with fluid while gross ascites were by marked abdominal distension. Upper GI bleeding was manifested by hematemesis, vomitus of red blood or "coffee-grounds" material; melena, black, tarry stool and /or endoscopy finding of upper GI bleeding.^3^

Child-Pugh classification of the severity of liver disease was based according to the degree of ascites, serum concentrations of bilirubin and albumin, prothrombin time, and degree of encephalopathy. A total Child-Pugh score of 5 to 6 is considered Child-Pugh class A (well-compensated disease); 7 to 9 is class B (significant functional compromise); and 10 to 15 is class C (decompensated disease).

Data were entered in Microsoft Excel Version 2010 and analyzed using IBM SPSS Statistics version 16.0. Point estimate and 95% CI were calculated.

## RESULTS

Among 157 patients admitted, CLD with ascites, SBP was seen in 46 (29.29%) (22.17-36.41, 95% CI). The mean age was 51.5±11.28 years (Table 1).

**Table 1:** Demographics parameters (n= 46).

Parameters	n (%)
**Gender**	
Male	34(73.91)
Female	12 (26.09)

**Figure 1:**
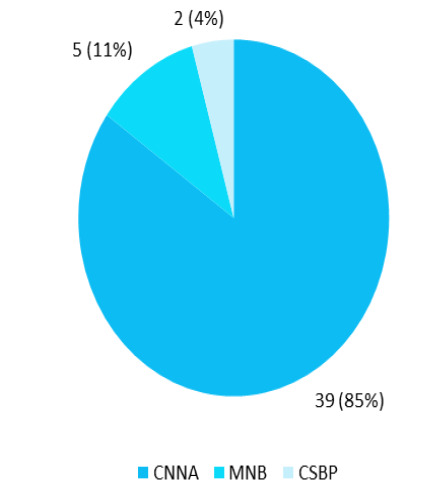
Classification of spontaneous bacterial peritonitis (n= 46).

The SBP patients were classified according to culture positivity and ANC count in ascitic fluid. Most cases were culture-negative neutrocytic ascites type 39 (84.78%) patients (Figure 1).

A total of 7 (15.22%) of patients had growth in ascitic fluid with *Klebsiella* species in 3 (6.52%), *Escherichia coli* in 2 cases (4.35%), *Streptococcus pneumonia* and *Acinetobacter* in 1 (2.17%) each.

The most common presenting symptom in patients of SBP was pain abdomen 23 (50%), followed by upper gastrointestinal bleed 19 (41.30%). Abdominal pain was seen in 29 (63%) (Table 2).

**Table 2:** Frequency of presenting signs and symptoms (n= 46).

Signs and symptoms	n (%)
Fever	9 (19.57)
Abdominal pain	29 (63.04)
**Ascites**	
Moderate	8 (17.39)
Gross	38 (82.61)
Encephalopathy	16 (34.78)
UGI bleeding	19 (41.30)

The mean ascitic fluid total protein was 2±0.86 (Table 3).

**Table 3:** Ascitic fluid parameters (n= 46).

Parameters	Mean±SD (gm/dl)
Ascitic fluid total protein	2±0.86
Ascitic fluid albumin	1±0.33
SAAG	1.5±0.47

Among the 46 patients, 39 (84.78%) were of Child Pugh Class C (Table 4).

**Table 4:** Classification according to Child Pugh Class (n= 46).

Class B	7 (15.22)
Class C	39 (84.78)

## DISCUSSION

The prevalence of SBP in admitted CLD patients with ascites was 29.3% which was high when compared to a similar study done in other tertiary centres of Nepal,^5^ but similar to other studies done in Nepal.^10^ In this study, ascitic fluid culture grew organism in 15% of SBP patients which was similar when compared to a study done in Nepal with culture positivity of 12.5%,^5^ but quite low in comparison to the expected 72-90% of culture positivity.^9^ It was probably due to prior intake of antibiotics by the patients. The most common organism was Gram-negative bacteria namely; *Klebsiella* sps. and *E. coli* which was similar to previous studies.^11,12^ In the current investigation, abdominal pain was significantly more common in SBP patients, occurring in 29 (63%) cases followed by upper gastrointestinal bleeding 19 (41%), hepatic encephalopathy 16 (34.8%) and fever 9 (19%) which was similar to another study.^13^ SBP can occur without abdominal pain also.^10^ In this research, the frequency of upper gastrointestinal bleeding, hepatic encephalopathy and gross ascites were similar in the two groups probably because coagulopathy, portal hypertensive gastropathy, varices and electrolyte imbalance were present in most the cases as most of them were of Child-Pugh Class C.

In this study, there was no difference between patients with SBP and patients without SBP regarding total leukocyte count, haemoglobin, and platelets count which was in agreement with other studies.^14^ SGOT level was higher than SGPT in the SBP group which was similar to other studies.^15^ On comparison between the two groups, SGPT level was significantly more raised in patients with SBP; which was similar to another study.^16^ It was probably due to ongoing injury to the liver due to inflammation caused by SBP.^17^

On comparison of total bilirubin, serum albumin, INR, and serum creatinine, there was no significant difference between the groups which was similar to other studies.^15^ Also no significant difference was found regarding SGOT, Na, K, ascitic fluid protein, albumin and SAAG.

The limitation of this study was that the sample size of the groups compared was not equal. This may lead to many variables failing to achieve statistical significance. This study was performed at a tertiary care hospital which receives patients with relatively advanced diseases and so it may not be comparable in primary care settings.

## CONCLUSIONS

The prevalence of spontaneous bacterial peritonitis was found to be higher than the other studies done in similar settings. Abdominal pain when present suggests SBP but can occur without it. So, in every healthcare facility either primary or tertiary, diagnostic paracentesis should be done on admission if the patient complains of abdominal pain. In addition, it should be done even if the patient is asymptomatic. Clinicians should be aware of this fact and have a high degree of suspicion for early diagnosis and treatment.

## References

[1] Cheemerla S, Balakrishnan M (2021). Global epidemiology of chronic liver disease.. Clin Liver Dis (Hoboken)..

[2] Jalan R, Fernandez J, Wiest R, Schnabl B, Moreau R, Angeli P (2014). Bacterial infections in cirrhosis: a position statement based on the EASL Special Conference 2013.. J Hepatol..

[3] Bacon BR, Jameson J, Fauci AS, Kasper DL, Hauser SL, Longo DL, Loscalzo J (2018). Harrison's Principles of Internal Medicine.

[4] Paul K, Kaur J, Kazal HL (2015). To study the incidence, predictive factors and clinical outcome of spontaneous bacterial peritonitis in patients of cirrhosis with ascites.. J Clin Diagn Res..

[5] Maskey R, Karki P, Ahmed SV, Manandhar DN (2011). Clinical profile of patients with cirrhosis of liver in a tertiary care hospital, Dharan, Nepal.. Nepal Med Coll J..

[6] Piano S, Brocca A, Mareso S, Angeli P (2018). Infections complicating cirrhosis.. Liver Int..

[7] Fernandez J, Ruiz del Arbol L, Gomez C, Durandez R, Serradilla R, Guarner C (2006). Norfloxacin vs ceftriaxone in the prophylaxis of infections in patients with advanced cirrhosis and hemorrhage.. Gastroenterology..

[8] Tito L, Rimola A, Gines P, Llach J, Arroyo V, Rodes J (1988). Recurrence of spontaneous bacterial peritonitis in cirrhosis: frequency and predictive factors.. Hepatology..

[9] Pande R, Khatri R, Nepal N, Pande PR (2009). Study of frequency of spontaneous bacterial peritonitis in patients with alcoholic liver cirrhosis with ascites.. Postgrad Med J NAMS.

[10] Syed VA, Ansari JA, Karki P, Regmi M, Khanal B (2007). Spontaneous bacterial peritonitis (SBP) in cirrhotic ascites: a prospective study in a tertiary care hospital, Nepal.. Kathmandu Univ Med J (KUMJ)..

[11] Biggins SW, Angeli P, Garcia-Tsao G, Gines P, Ling SC, Nadim MK (2021). Diagnosis, evaluation, and management of ascites, spontaneous bacterial peritonitis and hepatorenal syndrome: 2021 Practice Guidance by the American Association for the Study of Liver Diseases.. Hepatology..

[12] Mattos AA, Wiltgen D, Jotz RF, Dornelles CMR, Fernandes MV, Mattos AZ (2020). Spontaneous bacterial peritonitis and extraperitoneal infections in patients with cirrhosis.. Ann Hepatol..

[13] Ribeiro TC, Chebli JM, Kondo M, Gaburri PD, Chebli LA, Feldner AC (2008). Spontaneous bacterial peritonitis: How to deal with this life-threatening cirrhosis complication?. Ther Clin Risk Manag..

[14] Lesinska M, Hartleb M, Gutkowski K, Nowakowska-Dulawa E (2014). Procalcitonin and macrophage inflammatory protein-1 beta (MIP-1ß) in serum and peritoneal fluid of patients with decompensated cirrhosis and spontaneous bacterial peritonitis.. Adv Med Sci..

[15] Siddiqi AI, Siddiqeh M, Mehmood A, Siddiqui AM (2007). Alanine aminotransferase/aspartate aminotransferase ratio reversal and prolonged prothrombin time: a specific indicator of hepatic cirrhosis.. J Ayub Med Coll Abbottabad..

[16] Elshayeb E.I., Badr M.H., Abdu Elgayed E.M., Nor EI dean AS (2019). Serum and ascitic procalcitonin as a marker for early diagnosis of spontaneous bacterial peritonitis.. The Egyptian Journal of Internal Medicine..

[17] Moreau R, Gao B, Papp M, Banares R, Kamath PS (2021). Acute-on-chronic liver failure: A distinct clinical syndrome.. J Hepatol..

